# Intrinsically disordered protein, DNA binding with one finger transcription factor (*OsDOF27*) implicates thermotolerance in yeast and rice

**DOI:** 10.3389/fpls.2022.956299

**Published:** 2022-07-29

**Authors:** Nishu Gandass, Kajal, Prafull Salvi

**Affiliations:** Department of Agriculture Biotechnology, National Agri-Food Biotechnology Institute, SAS Nagar, India

**Keywords:** intrinsically disorder proteins, heat stress response, DOF-transcription factor, crop improvement, transcriptional regulation

## Abstract

Intrinsically disorder regions or proteins (IDRs or IDPs) constitute a large subset of the eukaryotic proteome, which challenges the protein structure–function paradigm. These IDPs lack a stable tertiary structure, yet they play a crucial role in the diverse biological process of plants. This study represents the intrinsically disordered nature of a plant-specific DNA binding with one finger transcription factor (DOF-TF). Here, we have investigated the role of *OsDOF27* and characterized it as an intrinsically disordered protein. Furthermore, the molecular role of *OsDOF27* in thermal stress tolerance has been elucidated. The qRT-PCR analysis revealed that *OsDOF27* was significantly upregulated under different abiotic stress treatments in rice, particularly under heat stress. The stress-responsive transcript induction of *OsDOF27* was further correlated with enriched abiotic stress-related *cis*-regulatory elements present in its promoter region. The *in vivo* functional analysis of the potential role of *OsDOF27* in thermotolerance was further studied in yeast and *in planta*. Ectopic expression of *OsDOF27* in yeast implicates thermotolerance response. Furthermore, the rice transgenic lines with overexpressing *OsDOF27* revealed a positive role in mitigating heat stress tolerance. Collectively, our results evidently show the intrinsically disorderedness in *OsDOF27* and its role in thermal stress response in rice.

## Introduction

Rice is one of the most important cereal crops as it is consumed by one-third of the global population (Fornasiero et al., 2022). Despite an enormous rise in rice cultivation, its productivity is severely compromised by different environmental stresses, including extreme temperature, water scarcity, salinity stress, heavy metal stress, etc. Besides, the dwindling agrarian land and continuously growing population further escalated the gap between its demand and supply (Rasheed et al., 2020). To combat the stressful conditions, diverse molecular responses are instigated at the cellular level, which include signaling cascade, phytohormonal regulation, altered gene expression, transcriptional regulation, and post-transcriptional and post-translational modifications (Singh and Jwa, 2013; Cohen and Leach, 2019; Kaur et al., 2021; Manna et al., 2021; Salvi et al., 2021b, 2022). Transcriptional control involves the regulation mediated *via* transcription factors (TFs) and transcriptional regulators (TRs). TF bind to *cis*-elements present in the promoter region of the target gene while TR indirectly regulates the gene expression by modulating the DNA—protein interaction through chromatin remodeling (Zheng et al., 2016; Chandran et al., 2019; Manna et al., 2021). In rice, about 2,048 gene are predicted to encode for TFs, whereas 328 as transcriptional regulators (Kikuchi et al., 2003; Pérez-Rodríguez et al., 2010). Similarly, about 6% of the total Arabidopsis genome is assigned to encode for TF (Riechmann et al., 2000). Overall, the TFs are ascribed to perform diverse cellular functions. Amongst them, several TF families are known to play general functions, while some of the TF families belong to a plant-specific clade. Generally, the plant-specific TFs orchestrate the regulation of gene networks associated with processes that are specific to plants, such as seed germination and maturation, vasculature formation, photosynthesis, stomatal regulation, etc. (Hrmova and Hussain, 2021; Wani et al., 2021; Strader et al., 2022). Amongst a large number of TFs families, TCP, AP2, and DOF-TF are documented as plant-specific TFs (Manna et al., 2021).

The DNA binding with one finger transcription factor family belongs to a plant-specific clade of TF which is structured as a Cys2/Cys2 zinc finger-TF (Yanagisawa, 2002). As DOF-TFs are exclusively present in plants, they are widely known to regulate diverse biological events that are specific to the plant system, such as abiotic stress, vasculature formation, photoperiodic flowering, seed germination, seed development, circadian cycle, phytochrome signaling, nitrogen use efficiency, etc. (Shigyo et al., 2007; Zou et al., 2013; Gupta et al., 2015; Corrales et al., 2017; Manna et al., 2021). The number of DOF-TF varies greatly across the species, for instance, there are 37 DOF-TF encoding genes in *Arabidopsis* (Lijavetzky et al., 2003), 34 in tomato (Cai et al., 2013), 31 in wheat (Shaw et al., 2009), 60 in apple (Zhang et al., 2018), 74 in banana (Dong et al., 2016), 41 in poplar (Yang et al., 2006), and 78 in soybean (Guo and Qiu, 2013) to name a few. In general, the DOF-TF is a 200–400 aa long protein with a highly conserved DNA binding motif that usually resides in the N-terminal and greatly divergent C-terminal region. The N-terminal DNA binding domain of all DOF-TF specifically binds to the 5'-[T/A]AAAG-3' (except AGTA in pumpkin) *cis*-regulatory element present in the promoter region of its target gene, while its C-terminal interacts with the RNA polymerase to modulate the gene (Kisu et al., 1995, 1998; Noguero et al., 2013). Previous reports have also shown that apart from the DNA-binding potential of the DOF-domain, it also participates in the protein–protein interaction and thus indicates its functional versatility (Yanagisawa, 2004; Waqas et al., 2020; Manna et al., 2021). DOF-TF interacts with different proteins, especially other TFs such as TCP, MYB, and bZIP, which hold a crucial role in many biological pathways. The first DOF-TF encoding gene was identified in maize in 1995 which binds to the CaMV35S promoter sequence (Yanagisawa, 1995). Several reports indicated the contribution of DOF-TFs in biotic and abiotic stress responses. In tomatoes, SlDOF22 negatively regulates the ascorbic acid content. Besides, in RNAi lines of *SlDOF22*, the *SlSOS1* gene was down-regulated, which results in decreased salinity stress tolerance (Cai et al., 2016). The overexpression of *GhDOF1* enhances the salt and cold stress response in cotton (Su et al., 2017). Corrales et al. (2017) showed that the expression level of cycling DOF factor 3 (*CDF3*) is triggered by abiotic stress conditions including salt, heat, drought, and ABA treatment, and its overexpression lines exhibited improved stress tolerance while RNAi lines displayed reduced stress tolerance. Several reports highlighted that the transcription factors are profuse with the intrinsically disordered region (IDR), which offers functional plasticity and versatility to the TFs (Liu et al., 2006; Brodsky et al., 2020; Salladini et al., 2020). Intrinsically disordered proteins are characterized as proteins with an enriched disordered region and devoid of an ordered three-dimension structure, yet they are capable to execute diverse cellular functions (Jung et al., 2011; Sun et al., 2013). As compared to prokaryotes, eukaryotic proteomes are known to possess higher disordered proteins. This aspect of abundant IDR in eukaryotes also indicated the correlation of IDR with complexity at the functional and organism levels (DeForte and Uversky, 2017). These IDRs emerged as a new field in structural biology which are largely studied in the animal system. However, due to their central role in the regulation of metabolic and cell signaling processes, they are attaining much attention in plant science as well (Zamora-Briseño et al., 2021). Here, we aimed to explore the role of an *OsDOF27* using diverse *in-silico* molecular and functional analyses. In our analysis, we found the *OsDOF27* protein to be highly disordered in nature and appeared to play an important role in thermal stress response in rice.

## Materials and methods

### Plant materials, treatment, and bacterial strain

Rice (*Oryza sativa*) seeds (var. Taipei-309) grown at the National Agri-Food Biotechnology Institute, Mohali (Punjab) were used in this study for genetic transformation and stress experiments. For the genetic transformation of rice, embryogenic calli were used (Thakur et al., 2022). For stress imposition on rice seedlings, 7-day-old rice seedlings were challenged to different abiotic stressors such as heat (42°C), salt (200 mM), cold (4°C), methyl viologen (20 μM), polyethylene glycol (PEG; 20%) for 12 h. After 12 h of stress, the seedling was immediately used for expression profiling or snap frozen and stored at −80°C for later use (Yokotani et al., 2009). For cloning experiments, the *Escherichia coli* DH5α strain was used, while *Agrobacterium tumefaciens* LBA-4404 was used for rice transformation. Yeast strain (INVSc1) was used as a host strain for the yeast expression system to study thermotolerance response in yeast.

### Molecular cloning

For molecular cloning of *OsDOF27 (LOC_Os10g35300)*, we used gateway-based cloning as per the manufacturer's instructions. Initially, the *OsDOF27* was cloned in pENTR™/D-TOPO™ vector and the positive constructs were confirmed by Sanger sequencing. The sequenced confirmed entry clone was then used for its subcloning to the destination vectors like pANIC6B (CD3-1708), pSITEYFP3CA (CD3-1638), and pDEST52 (pYES2-DEST52 Gateway™ destination vector) using LR Clonase-II. The primers used for this study are enlisted in Supplementary Table 1.

### Agroinfiltration of *N. benthamiana* for subcellular localization study

The subcellular localization study was conducted by agroinfiltration according to our method described previously (Thakur et al., 2021). Briefly, *Agrobacterium* cells harboring positive transformant of pSITEYFP3CA were grown in LB medium with selection markers at 28°C for 24 h. The plasmid with 35S-YFP was used as the positive control. The *Agrobacterium* cells were then pelleted down by centrifugation, mixed with resuspension buffer, and incubated for 6 h prior to infiltration. The mix was then infiltrated into the abaxial surface of 6-week-old *N. benthamiana* leaves with a needleless syringe (1 ml). The plants were covered and kept in dark for 12 h and then transferred to the growth chamber at 24°C ± 1 with a 16/8 h photoperiod. The leaf sample was collected after 48 h for microscopy.

### DAPI staining and confocal microscopy

The nuclear localization of *OsDOF27* protein was confirmed by colocalizing it with 4, 6-diamidino-2-phenylindole (DAPI) that specifically stains the nucleus. Briefly, the leaf sample was cut and washed by dipping (for 5 s) with 1X phosphate-buffered saline (PBS, pH-7.5) containing 0.5% Triton-X (which aids the DAPI staining). After a gentle wash (for 5–10 s) in 1X phosphate-buffered saline (PBS, pH-7.5; without Triton-X), the leaf sample was incubated in DAPI solution (15 μg mL^−1^ DAPI prepared in 1 × PBS) for 20 min. The fluorescent signals were detected using a Carl Zeiss confocal laser scanning microscope (LSM880; Karl Zeiss, Jena, Germany) with an oil immersion objective (Thakur et al., 2021).

### QPCR analysis

Total RNA was isolated using the TRIzol reagent (Sigma) and reverse transcribed into cDNA using iScript™ cDNA Synthesis Kit (BioRad) according to the manufacturer's instruction. A no template control was incorporated as a negative control in each assay. For normalization, two endogenous reference genes (Ubiquitin-5 and EF1α) were used in each assay. The gene-specific primers and reference genes used for the qRT-PCR were previously described and validated.

### Yeast thermo-tolerance assay

To assess the thermotolerance assay in the yeast system, we cloned *OsDOF27* in the yeast expression vector (pDEST52 vector) using gateway-based cloning (Invitrogen). The construct pDEST52:*OsDOF27* was then mobilized to yeast strain (INVSc1) through PEG-lithium acetate-based transformation. Yeast spot assay and growth curve analysis were performed to determine the thermotolerance of the yeast cell harboring pDEST52:*OsDOF27*. The yeast cells were allowed to grow in a YEB medium till the mid-exponential phase with *A*__60_0_ 0.5 (about 1 × 10^7^ cells ml^−1^). The cell density was then adjusted to 0.2 (A_600_) for spot assay. Spot assay was conducted by spotting a serial dilution yeast culture with equal cell density (A_600_ 0.2), and a 5 μl spot was placed on the plate. An empty-vector-transformed INVsc1 strain was used in each case as a control for the stress experiment. For unstressed conditions, the plate was incubated at 30°C, while to determine the thermotolerance potential, the plate was incubated at 42°C for 8 h and then moved to 30°C. The yeast cell growth on the plate was monitored closely and photographed. For growth curve analysis, the initial cell density of the yeast cell harboring pDEST52:*OsDOF27* and the empty vector was kept similar (OD = 0.2 A), and the growth was observed and plotted against time. Each assay was repeated three times, with at least four biological replicates (Salvi et al., 2021a).

### *In silico* analysis of *OsDOF27* for IDR analysis and stability curve

The *OsDOF27* sequence was obtained from the Rice Genome Annotation Project database (http://rice.uga.edu/). The disorder propensity was determined with different software such as http://www.pondr.com/, https://iupred.elte.hu/, and http://sparks-lab.org/server/SPOT-disorder/index.php. The compositional profiler was used to assess the enrichment or depletion of the amino acid composition of *OsDOF27* against SwissProt52. The amino acid composition was color-coded for disorders and sorted by the differences observed (http://www.cprofiler.org/cgi-bin/profiler.cgi; Vacic et al., 2007). For generating the sequence, annotated Das-Pappu phase diagram Classification of Intrinsically Disordered Ensemble Regions, CIDER, http://pappulab.wustl.edu/CIDER/analysis/) was used to evaluate the parameters associated with disordered protein sequences (Kyte and Doolittle, 1982; Das and Pappu, 2013; Holehouse et al., 2015). The prediction of secondary structures such as alpha helix, beta turns, extended strand, and the random coil was conducted by the online tool Self-Optimized Prediction Method with Alignment (SOPMA, https://npsa-prabi.ibcp.fr/cgi-bin/npsa_automat.pl?page=/NPSA/npsa_sopma.html; Geourjon and Deléage, 1995). To assess parameters like the standard heat capacity of folding (ΔCp), the standard enthalpy of folding (ΔHm) at melting temperature, Tm (melting temperature), the protein sequence of *OsDOF27* was subjected to an online tool SCooP tool (http://babylone.3bio.ulb.ac.be/SCooP/index.php). The curve for the change in standard free energy of folding with respect to the temperature was assessed by protein stability curve prediction (Pucci and Rooman, 2014; Pucci et al., 2017).

### Agrobacterium-mediated rice transformation

*Agrobacterium*-mediated rice transformation was performed as per our previous report (Thakur et al., 2022). Briefly, *Agrobacterium tumefaciens* strain LBA4404 harboring pANIC6C*OsDOF27* (*LOC_Os10g35300*) was used for the genetic transformation of rice. For rice transformation, embryogenic calli were co-cultivated with *Agrobacterium* culture for 36 h and further washed and selected for hygromycin resistance. After three rounds of selection, the callus was transferred to regeneration media. Plants with developed roots and shoots were transferred to a hydroponics medium and subsequently hardened and were grown to maturity. The offspring of the primary transformants were propagated by selfing and T2 generation seeds were used for further experiments.

### Stress treatment

For stress experiments, the seeds of three independent transgenic lines (Ubi:*OsDOF27*-OE1, OE-2, and OE-5), wild type (WT), and vector control (VC) were exposed to high temperature. Seed germination was assessed on two layers of filter paper (Whatman No. 1) moistened with autoclaved double-distilled water (ddH_2_O). For non-stressed conditions, seed germination was evaluated at 28 ± 1°C while for heat stress, the seeds were pre-exposed to high temperature (45°C) for 12 h. The germination rate was determined by monitoring the germination score after 72 h. Gemination assay was conducted in triplicate with n=30 seeds per treatment in each experimental replicate. Seeds that exhibited radicle protrusion were scored as germinated. For stress imposition at the seedling stage, rice transgenic and control plants were germinated under non-stressed/control conditions at 28 ± 1°C with a photoperiod of 16 h-light/8 h-dark cycle (300–400 μmol photons m^−2^s^−1^). Two-week-old plants were challenged to heat stress by transferring them to 45°C for 24 h and then transferred to 28 °C, while for non-stressed (NS) conditions, plants were continuously grown at 28°C.

## Results

### Gene expression analysis of rice *OsDOF27* during abiotic stress conditions

**Figure 1:**
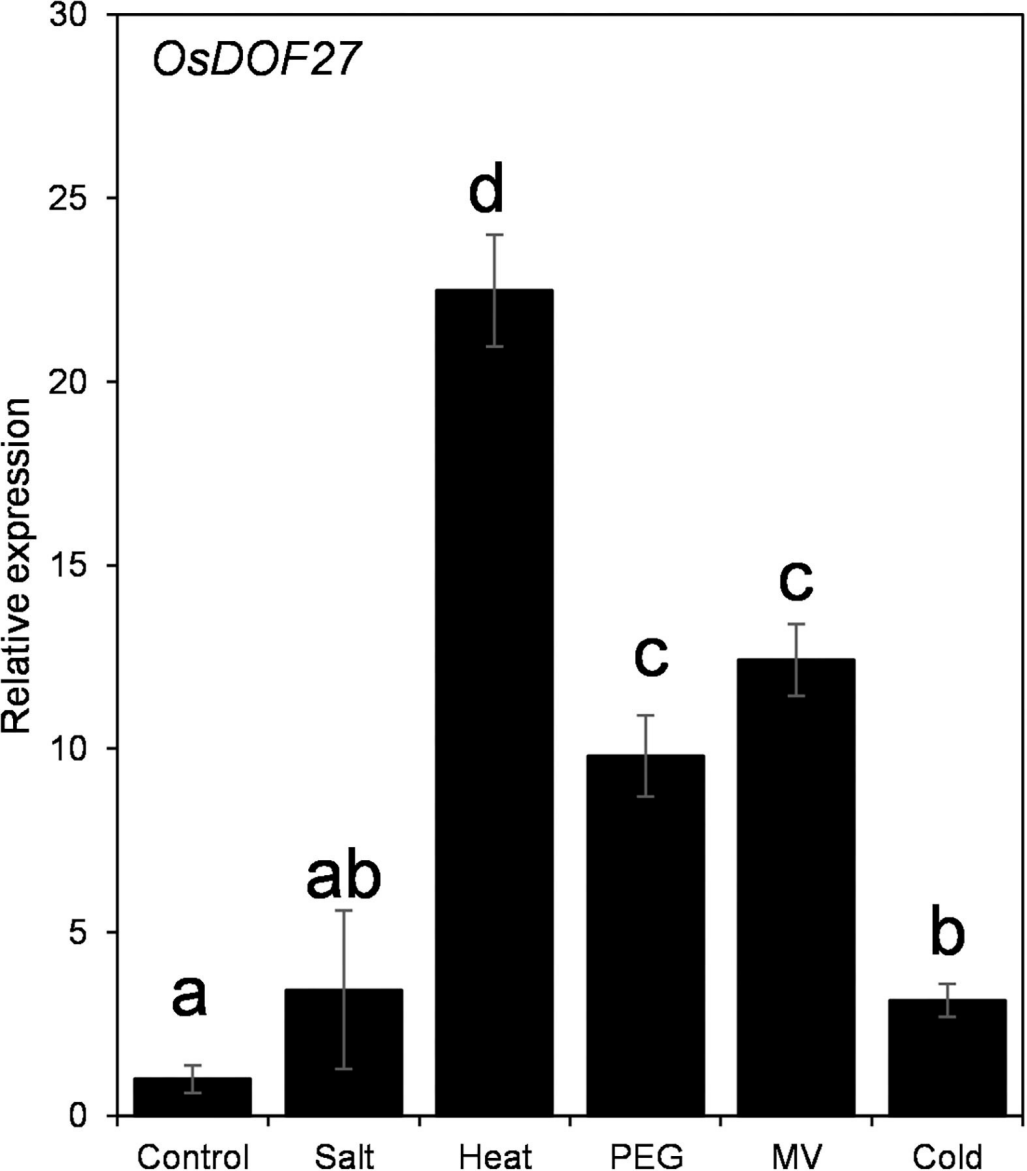
qRT-PCR for expression analysis of *OsDOF27* under different abiotic stress conditions including salt, heat, PEG, methyl viologen, and cold. Seven-day-old rice seedlings were treated with abiotic stressors (20% PEG, 200 mM NaCl, heat (42°C), cold (4°C), and methyl viologen (20 μM). Total RNA was extracted, and cDNA was synthesized, followed by qPCR. Two endogenous controls (Ubiquitin-5 and EF1α) were used to normalize the relative expression value of *OsDOF27* followed by a calculation using the ^ΔΔ^*CT* method. The error bars represent the standard deviation of triplicate analysis. The significant difference in the mean of the values is indicated by different letters (α = 0.01).

To understand the biological function of DOF-TF in rice plant growth and development, we have investigated the expression of *DOF27* under abiotic stress treatments. For this, rice seedlings were challenged with abiotic stressors including heat, salt, cold, PEG, and methyl viologen, and the transcript level of *OsDOF27* was assessed using qRT-PCR. *OsDOF27* showed maximum induction in heat stress followed by oxidative and osmotic stress imposed by methyl viologen (paraquat) and PEG treatment, respectively (Figure 1). To get a molecular insight into the gene regulation of *OsDOF27*, we inspected the *cis*-regulatory elements present in the promoter region of *OsDOF27*. For this, the 1.5 KB promoter sequences of *OsDOF27* were analyzed using web-based tools i.e., PLACE database. The results revealed that the promoter regions were enriched with various stress and phytohormonal-related *cis*-regulatory elements indicating the stress-responsive expression of *OsDOF27*. Supplementary Table 2 showed the detailed *cis*-element present in the promoter region of *OsDOF27*.

### *DOF27* is an intrinsically disorder protein

**Figure 2:**
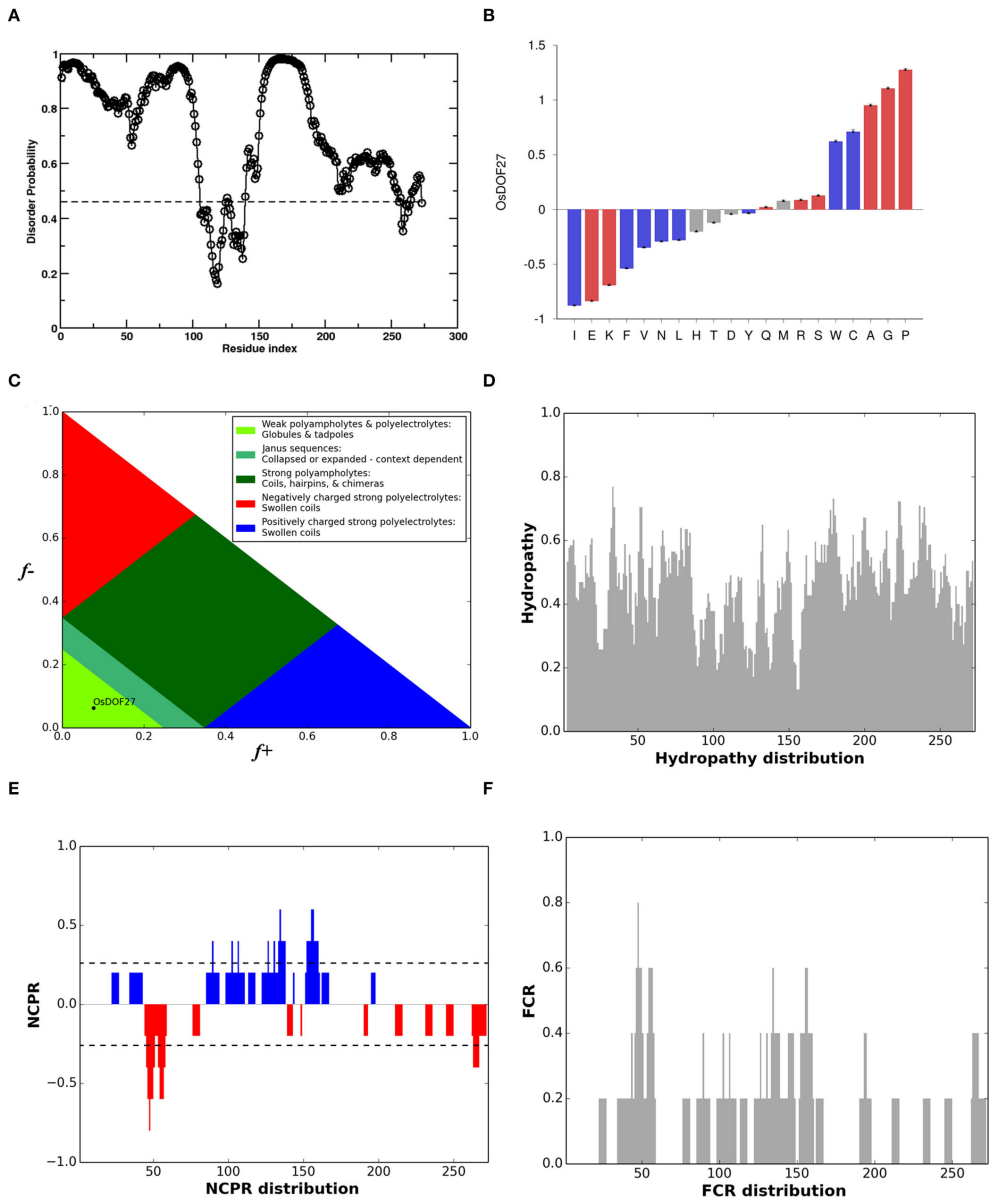
**(A)** Graph represents a prediction of disorder regions present in the protein sequence of *OsDOF27* based on the amino acid composition. Calculations were performed using the database of Protein Disorder (DisProt) software. A score over 0.5 indicates a high probability of disorder. **(B)** Depiction of compositional bias of *OsDOF27* to show enriched or depleted aa of *OsDOF27* against the SwissProt52 database. The amino acid composition was color-coded for disorder and sorted by the difference observed. The red and blue color bars indicate the disordered and ordered promoting amino acid, respectively, while the gray color-coded bar represents the neutral amino acid. **(C)** Analysis for diagram of states which represent the position of protein in graph drawn between the fraction of positive and negative charge, **(D)** Hydropathy plot (based on *Kyte-Doolittle hydrophobicity scale*), **(E)** NCPR (net charge per residue), **(F)** FCR (fraction of charged residue) of *OsDOF27* protein sequence.

Furthermore, while designing the primers for *OsDOF27*, we observed multiple repeats in its sequence. It prompted us to inspect the protein sequence of *OsDOF27*, which showed a biased composition for amino acids as around 40% of the total sequence comprises three amino acids i.e., proline (15.4%), glycine (14.7%), and alanine (11%). The amino acid composition of *OsDOF27* suggested the presence of the intrinsically disordered region. Therefore, to understand the sequence–structure relationship of *OsDOF27*, we inspected the protein sequence of *OsDOF27* for the presence of an intrinsically disordered region (IDR). The *OsDOF27* appears to be a highly disordered protein that was enriched in the IDR region, especially in N- and C- terminals. However, the DNA binding DOF-domain was found to be deficient in IDR sequences (Figure 2A). The compositional bias of *OsDOF27* was assessed and compared against the Swissport 52 database, which revealed that *OsDOF27* possessed an abundant subset of disordered promoting amino acids such as proline, glycine, alanine, serine, and arginine, while the order promoting residues, such as isoleucine, phenylalanine, valine, etc., were dramatically low in *OsDOF27* (Figure 2B). Additionally, we also conducted an evaluation of the *OsDOF27* protein sequence for parameters like FCR (fraction of charged residue), NCPR (net charge per residue), Hydropathy plot (based on *Kyte-Doolittle hydrophobicity scale*), and diagram of states which represent the position of protein in graph drawn between the fraction of positive and negative charge. As shown in Figure 2, the different characterized IDPs were observed as κ (kappa) (0.310), FCR (0.139), NCPR (0.015), and Hydropathy (4.184) as per Das et al. (2015; Supplementary Table 3). Furthermore, the secondary structure prediction of *OsDOF27* also showed that it comprises 24.91% of alpha-helix, 12.09% extended-strand, 7.69% beta-turn, and more than 50%, i.e., 55.31%, is random-coil (Supplementary Figure 1).

### Subcellular localization of *OsDOF27*

**Figure 3:**
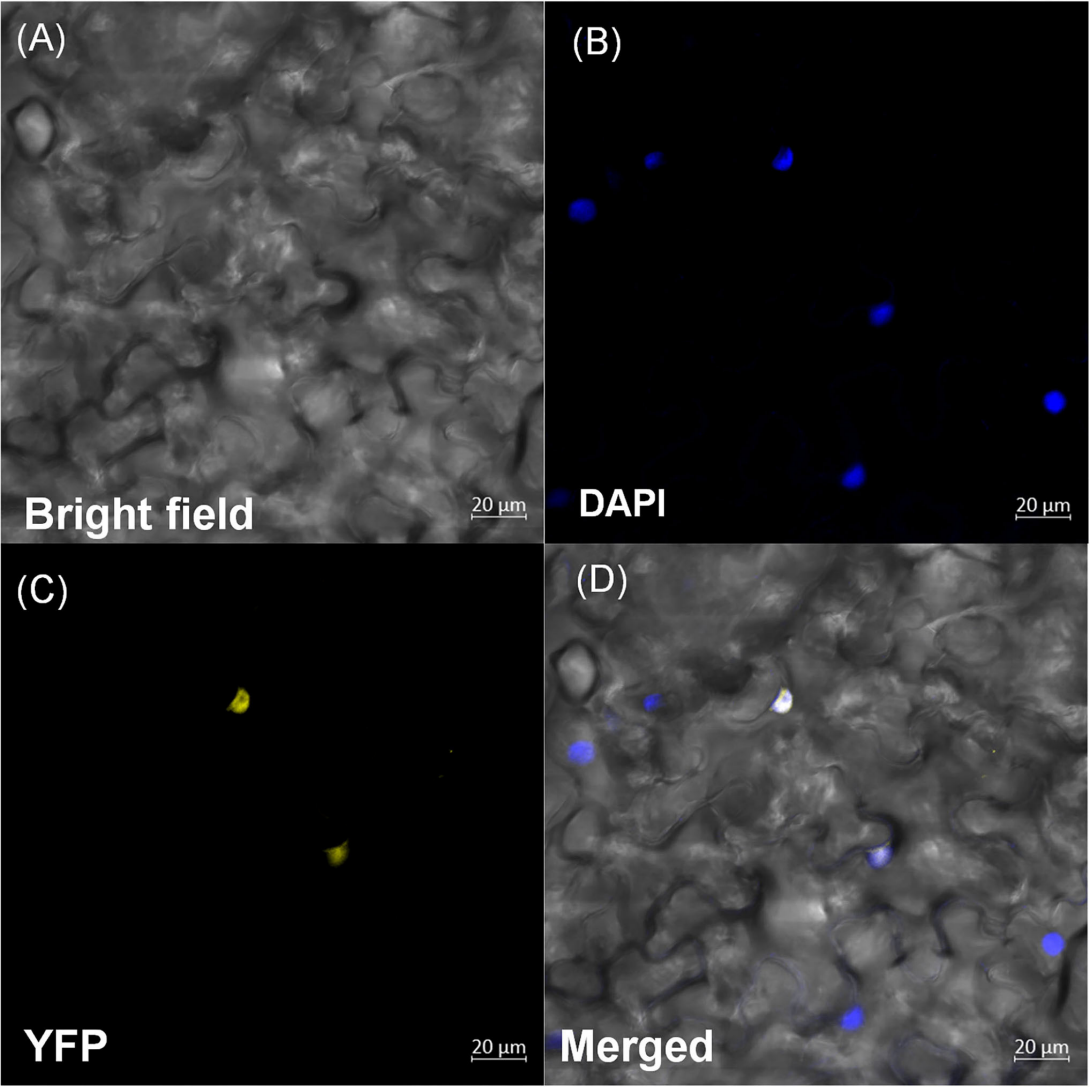
Subcellular localization of *OsDOF27* protein. Six-week-old Nicotiana leaves were syringe-infiltrated at the abaxial surface with *Agrobacterium* strain harboring pSITEYFP3CA:*OsDOF27* constructs. YFP fluorescence and differential interference contrast images and visible/GFP merged images are visualized by confocal microscopy. Scale bars = 20 μm. Confocal images of nicotiana leaves showing *OsDOF27* targeted to the nucleus, with **(A)** bright field; **(B)** DAPI; **(C)** YFP; **(D)** merged or overlay.

An *in silico* assessment of subcellular localization suggested the nuclear localization of *OsDOF27* (Supplementary Figure 2A). To examine the subcellular localization of *OsDOF27 in planta*, the CDS was cloned in the pSITE-YFP, and after sequence confirmation, the construct was transformed to *Agrobacterium*. The *Agrobacterium* strain harboring *OsDOF27* was used for agroinfiltration of transient transfection in *Nicotiana* leaves. After 2 days of agroinfiltration, the YFP signal was visualized using a confocal laser scanning microscope to track the subcellular localization of *OsDOF27*. The microscopic analysis revealed that *OsDOF27* was localized to the nucleus. The nuclear localization was further confirmed using DAPI staining, which specifically stains the nucleus. The YFP signal colocalized with DAPI, which confirms the nuclear localization of *OsDOF27* (Figure 3). However, in the control, fluorescence was detected in the entire cell, including the cytoplasm and nucleus (Supplementary Figure 2B).

### *OsDOF27* imparts thermotolerance in yeast

**Figure 4:**
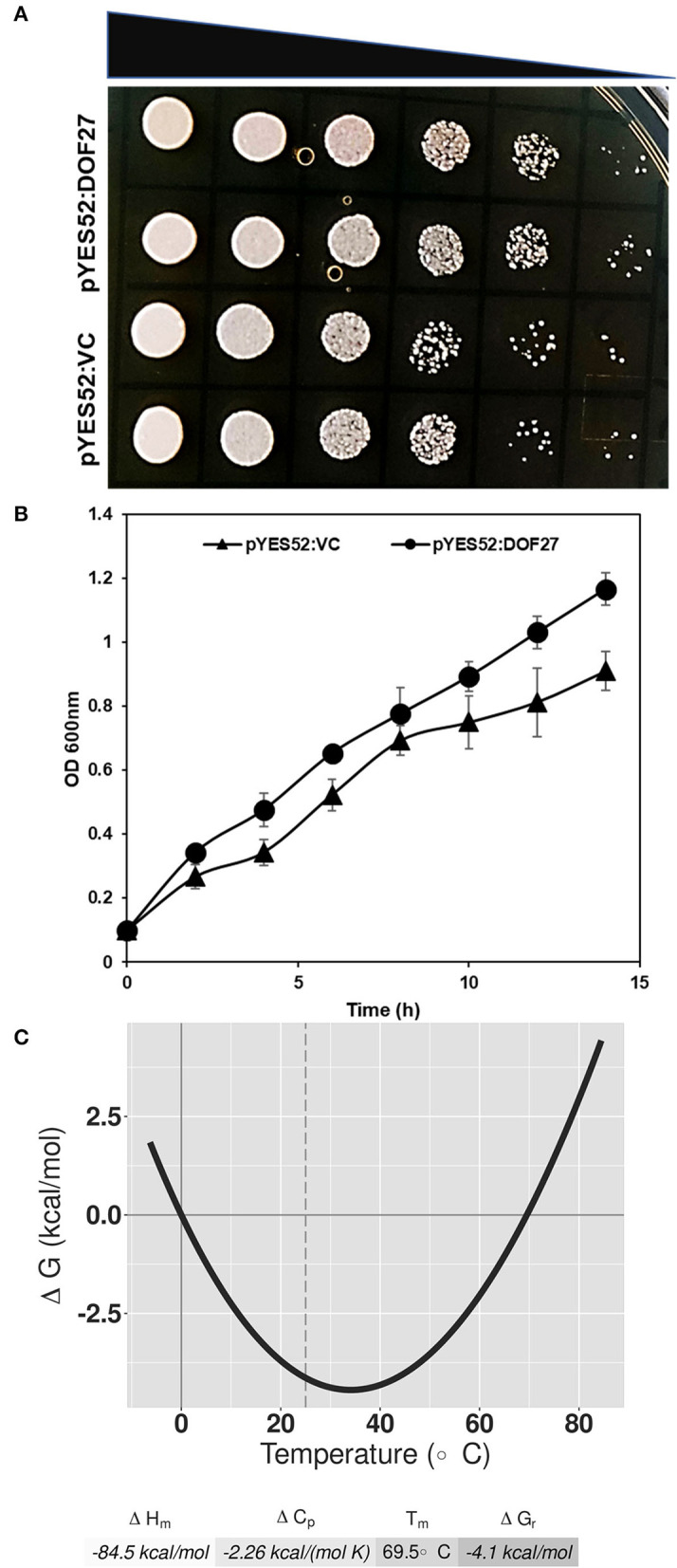
Thermotolerance potential of yeast is shown by **(A)** Spot assay which represents the comparative growth of yeast cells harboring pDEST52:*OsDOF27* and empty vector. Five microliters of each yeast sample (two colonies) were used for spot assay with 10-fold serial dilutions. **(B)** the curve depicts the growth of yeast cells harboring pDEST52:*OsDOF27* and empty vector under thermal stress. **(C)** The thermal stability curve represents the standard free energy of protein folding against the temperature.

As *OsDOF27* was highly induced under heat stress, we examined the thermotolerance potential of yeast cells harboring *OsDOF27*. For this, the *OsDOF27* was cloned in pDEST-52 in which the *OsDOF27* was regulated by gal4 promoter. After verifying the sequence of construct by Sanger sequencing, the construct (*pDEST-52-DOF27* or *pDEST-52-VC*) was mobilized to the yeast strain (INVSc1). We then examined the thermotolerance potential of yeast transformants using growth kinetics and the spot assay. The growth analysis indicated that *OsDOF27* ameliorates the thermal stress response of yeast cells expressing *OsDOF27* (Figures 4A,B). Additionally, in an *in silico* analysis based on the Gibbs-Helmholtz equation Δ*G(T)*= Δ*Hm[1–(T/Tm)]–*Δ*Cp[Tm–T*+*T Log(T/Tm)]*, the *OsDOF27* exhibited high Tm (melting temperature) of 69.5°C, which indicates its thermostability (Figure 4C). A *Tm* value indicates the temperature for a protein at which it gets denatured resulting in its functional impairment. For a protein to be thermally stable, it needs a more negative value for ΔH, which makes the ΔG value negative and thus more thermally stable.

### Overexpression of *OsDOF27* improves seed germination and stress response in rice transgenics

**Figure 5:**
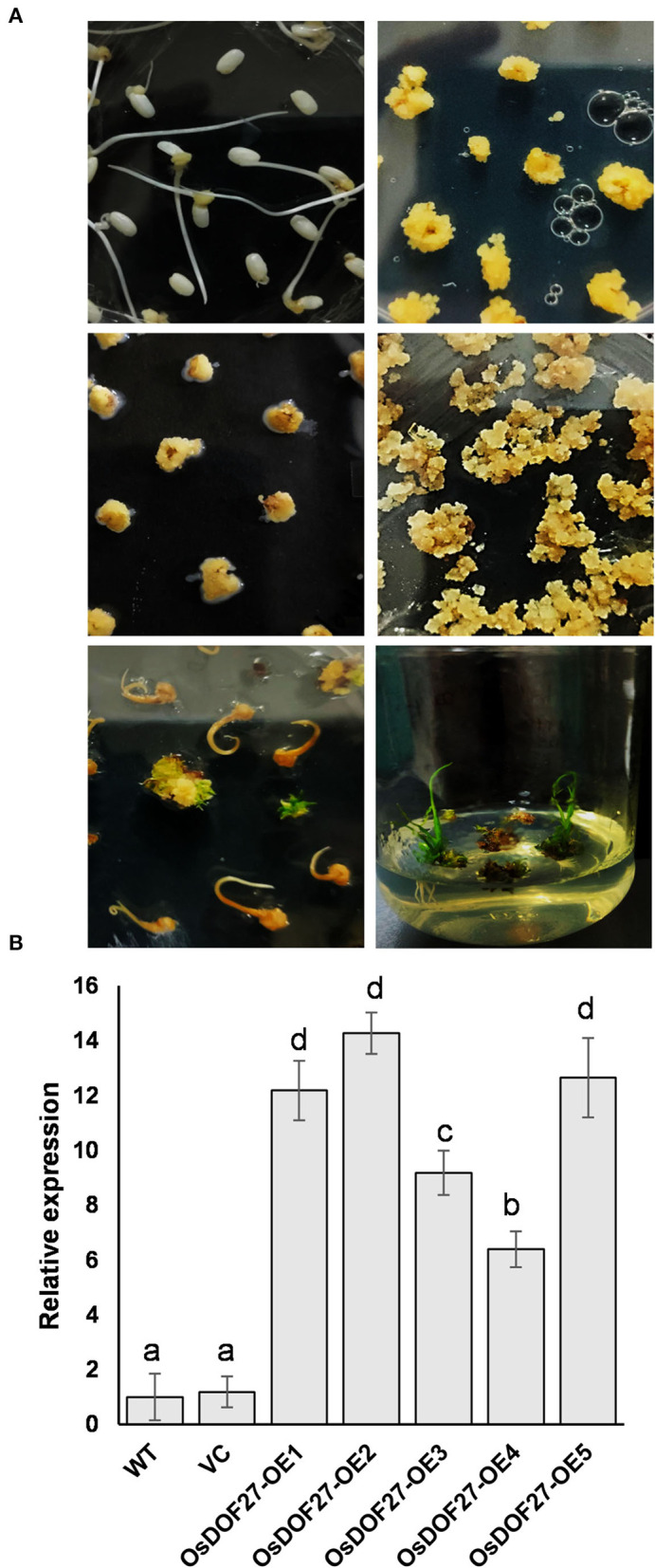
**(A)** Schematic representation of the *Agrobacterium*-mediated rice transformation of *OsDOF27*. **(B)** qPCR analysis of positive transformants. Total RNA was extracted from independent transgenic lines, and cDNA was synthesized followed by qPCR. Two endogenous controls (Ubiquitin-5 and EF1α) were used to normalize the relative expression value of *OsDOF27* followed by calculation using the ^ΔΔ^CT method. The error bars represent the standard deviation of triplicate analysis. The significant difference in the mean of the values is indicated by different letters (α = 0.01).

**Figure 6:**
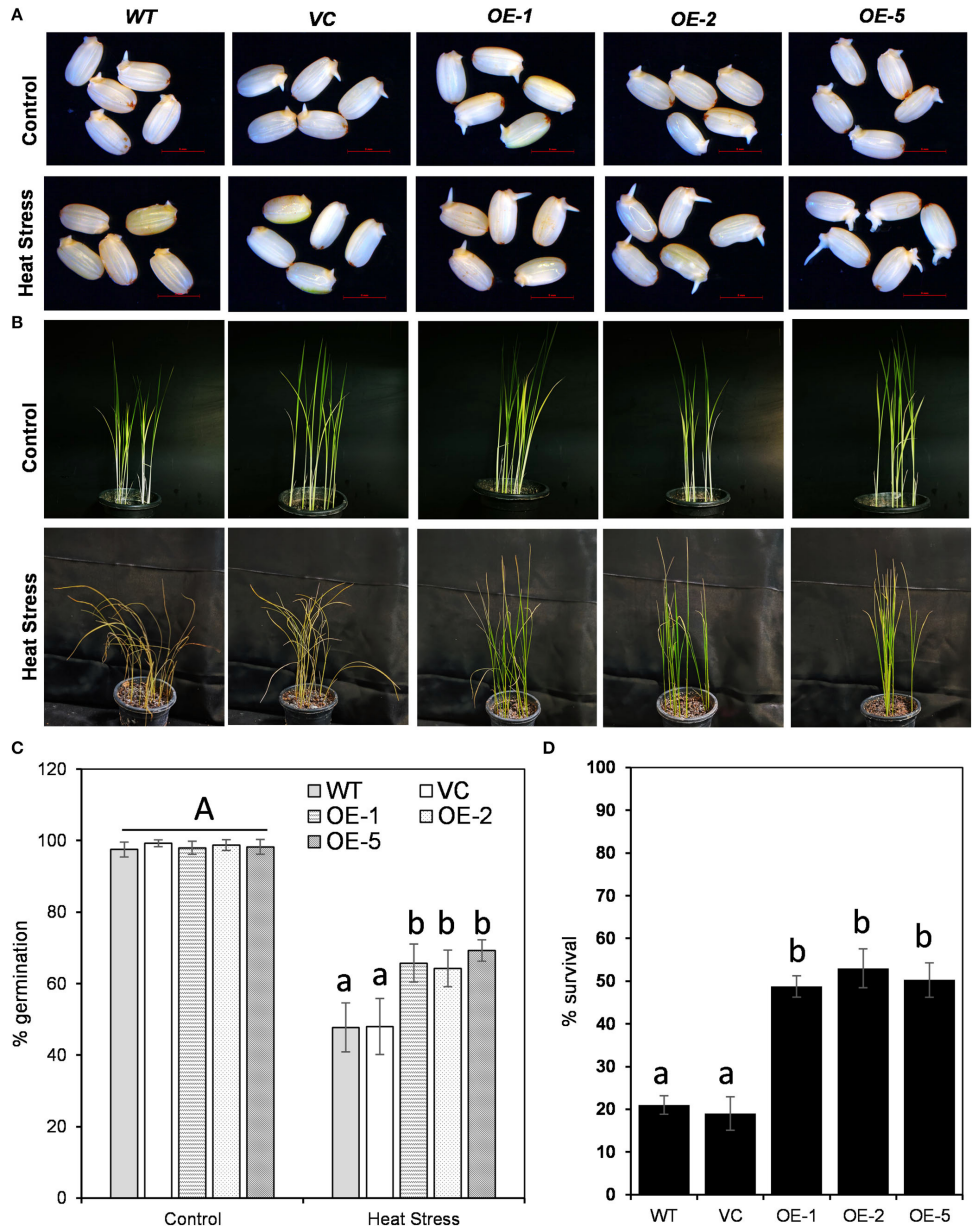
Comparative stress tolerance potential of transgenic lines overexpressing *OsDOF27* and its control counterpart (WT, wildtype plants, and VC, vector control plants) at **(A)** germination stage and **(B)** seedling stages. **(C)** quantitative analysis of percent germination and **(D)** percent survival of transgenic lines overexpressing *OsDOF27* in comparison to WT and VC under heat stress treatment. The error bars represent the standard deviation of triplicate analysis. The significant difference in the mean of the values is indicated by different letters (α = 0.01).

For functional analysis of *OsDOF27*, we generated rice transgenic lines overexpressing *OsDOF27* using *Agrobacterium*-mediated rice transformation (Figure 5A). The enhanced transcript level of *OsDOF27* in transgenic lines was assessed through qRT-PCR analysis (Figure 5B). Three independent transgenic lines (T2 lines) with enhanced expression of *OsDOF27* were then assessed for heat stress tolerance as qRT results of *OsDOF27*, and yeast thermotolerance assay indicated its role in heat stress. For this, the transgenic lines were challenged with heat stress at the germination stage and the seedling stage. First, the germination potential of three independent lines of T2 transgenic seed of *OsDOF27*-OE with enhanced *OsDOF27* expression along with the WT counterpart was examined. The transgenic seeds of the overexpression line showed better germination potential as compared to wild-type during heat stress (Figure 6A). While in the non-stressed condition, the transgenic and WT seeds did not show any significant difference in the germination assay. At the vegetative stage, when the seedlings were exposed to 45°C for 24 h and then transferred to 28°C, all three transgenic lines of *OsDOF27-OE* recovered better in comparison to their control counterpart (WT and VC; Figures 6B,C). The survival rate of transgenic plants was almost double than that of wild-type counterparts (Figure 6D). Besides, under non-stressed conditions, *OsDOF27-OE* lines behaved similar to WT and VC and did not exhibit any significant differences.

## Discussion

In general, plants possess incredible molecular plasticity, which supports their growth and development during their inevitable exposure to abiotic stresses. Some plants have an intrinsic ability for higher stress tolerance than others, such tolerance is dependent on different genetic and environmental factors of the plant. The ability of plants to tolerate the stressful milieu is largely controlled by their highly complex yet regulated transcriptional process (Manna et al., 2021; Strader et al., 2022). The transcription factors play a crucial role by regulating the expression of their target gene and controlling almost all the biological processes, including seed maturation, growth and development, stress tolerance, and senescence to name a few (Yanagisawa, 2002; Dubos et al., 2010; Manna et al., 2021). Being a DNA binding protein, the role of TFs is largely known for regulating the transcription by binding to the promoter region of their target gene. However, in light of recent research developments in protein biology, intrinsically disorder proteins are attaining much focus. The IDPs challenge the sequence–structure–function paradigm, as IDPs do not possess a stable three-dimension structure. The IDP with molecular flexibility possesses the ability to interact with different interacting partners due to the altered conformation under different physiological conditions (Sun et al., 2013; Zamora-Briseño et al., 2021). IDP or the presence of IDRs in the protein facilitates functional versatility. Apparently, the presence of abundant IDP or IDR in plants explains the molecular plasticity that the plant might have acquired during the course of evolution to support their adequate growth and development (Pazos et al., 2013).

Here, in this study, we have the intrinsically disorder nature of DOF-TF. We characterize the molecular role of *OsDOF27* under heat stress using two important systems, that is yeast and rice. The *in silico* analysis showed that the *OsDOF27* is involved in different biological processes, which indicated its functional versatility (Supplementary Figure 3). The promoter region of *OsDOF27* was enriched in abiotic stress-responsive *cis*-regulatory elements, and the expression profile tested also showed that *OsDOF27* induces under abiotic stress. Besides, stress response, its promoter region is also enriched in *cis*-elements related to the response of phytohormones such as ABA, ethylene, and JA. The promoter region also showed a high propensity for the DOF-TF binding site, indicating its self-regulation or regulation by other members of the DOF-TF family (Supplementary Table 2). The ponder fit analysis indicated that *OsDOF27* is an IDP; however, the region encompassing the DOF domain (109–158 aa; Supplementary Figure 4) has a very low disorder region. Since the DOF domain is a characteristic domain of the DOF family with a highly conserved region, which is known as the DNA binding region, this domain needs to have an ordered promoting amino acid. The abundance of arginine (R), serine (S), alanine (A), glycine (G), and proline (P) amino acids in the *OsDOF27* indicated its discorded nature and thus its structural flexibility. Also, the predicted secondary structure of *OsDOF27* encompasses more than 50% protein as a random coil (55%) supporting the disorder region of the protein (Smith et al., 1996). The abundance of glycine and proline in the *OsDOF27* drives it entropically unpropitious to form ordered secondary structures and makes the polypeptide more disordered and flexible. As glycine (lack side chain) caters to flexibility, proline (constrained phi angle) is too rigid to facilitate the adequate secondary structure (Eyles and Gierasch, 2000; Rauscher et al., 2006; Muiznieks and Keeley, 2010) Being a transcription factor, *OsDOF27* localizes to the nucleus, also the similar subcellular localization for DOF proteins are reported previously (Corrales et al., 2017; Su et al., 2017). The *OsDOF27* imparts yeast thermotolerance, which was revealed by spot assay as well as growth curve analysis. The thermal stability curve also showed that *OsDOF27* possesses negative ΔG between temperatures of 20° and 40°C, indicating its stability index within the temperature range (Pucci et al., 2017; Yadav et al., 2021). Besides, the high negative value of Tm and less negative ΔH with negative ΔG signifies the thermal stability of *OsDOF27*. *In planta* analysis for thermotolerance also depicted that rice transgenic lines with enhanced expression of *OsDOF27* were more tolerant to heat stress exposure as compared to their wild-type counterpart. Transgenic lines displayed improved tolerance potential at germination and the seedling stage, which indicated the important role of *OsDOF27* under heat stress challenges. Similarly, JrDOF3 was found to have a positive role in heat stress tolerance by binding to the DOFCOREZM motif of JrGRAS2 to regulate its expression. JrGRAS2 in turn regulates the expression of heat shock proteins and thereby improves high temperature stress tolerance (Yang et al., 2018). In a recent transcriptomics study in cucumber, DOF transcription factor was found to be upregulated (Yu et al., 2022). A transcriptomic and proteomic analysis would be conducted to understand the candidate molecular targets of *OsDOF27*, which will allow us to get deeper insight and delineate the detailed molecular mechanisms of heat stress tolerance imparted by *DOF27* in rice. Besides, the protein–protein interaction analysis of *OsDOF27* is also underway to comprehend the integrated network of this intrinsically disorder protein and other biological functions.

## Data availability statement

The datasets presented in this study can be found in online repositories. The names of the repository/repositories and accession number(s) can be found in the article/Supplementary material.

## Author contributions

PS conceptualized the manuscript, supervised the authors, wrote the manuscript, analyzed the data, and edited the manuscript. NG, K, and PS performed the experiments. All authors contributed to the article and approved the submitted version.

## Funding

This work was supported by the research funding to PS from the Department of Science and Technology (DST), Government of India under the scheme of DST-INSPIRE Faculty Award (DST/INSPIRE/04/2018/003425), and SERB Core-Research-Grant (CRG/2021/000949), Government of India.

## Conflict of interest

The authors declare that the research was conducted in the absence of any commercial or financial relationships that could be construed as a potential conflict of interest.

## Publisher's note

All claims expressed in this article are solely those of the authors and do not necessarily represent those of their affiliated organizations, or those of the publisher, the editors and the reviewers. Any product that may be evaluated in this article, or claim that may be made by its manufacturer, is not guaranteed or endorsed by the publisher.
