# Supplementary material for: Intrinsically disordered protein, DNA binding with one finger transcription factor (OsDOF27) implicates thermotolerance in yeast and rice
Source: Front Plant Sci. 2022 Jul 29;13:956299. doi: 10.3389/fpls.2022.956299 (PMC9372624; doi:10.3389/fpls.2022.956299)
Supplement: Supplementary file 1 [file Data_Sheet_1.PDF]

Supplementary data

Intrinsically disordered protein, DNA Binding with One Finger transcription factor (OsDOF27) implicates thermotolerance in yeast and rice

Nishu Gandass[1], Kajal[1] and Prafull Salvi[1, *]

[1]Department of Agriculture Biotechnology, National Agri-Food Biotechnology Institute, SAS Nagar 140306, India;

nishukarate3198@gmail.com (N.G.);

mittalkajal003@gmail.com (K.);

salvi.prafull@gmail.com (P.S.)

*Correspondence

Dr. Prafull Salvi;

Department of Agriculture Biotechnology,

National Agri-Food Bio-technology Institute,

SAS Nagar 140306, India;

Email: salvi.prafull@gmail.com and prafull.salvi@nabi.res.in

Web of Science ResearcherID: https://publons.com/researcher/AAP-1007-2020/

ORCID: https://orcid.org/0000-0002-7834-8803

Table S1. Primer used for the cloning of genes and molecular analysis of transformants

Primer Name	Sequence (5’-3’)
**M13_F**	TGTAAAACGACGGCCAGT
**M13_R**	CAGGAAACAGCTATGACCATG
**OsDOF27_F (Gateway cloning)**	CACCATGGCTCCTCCCCAGCTTGCCGG
**OsDOF27_R (Gateway cloning)**	CGCGGCCGATGGCGCCGTGAC
**OsDOF27_F (qRT-PCR)**	ACGTCACGGATTAGCTCGAT
**OsDOF27_R (qRT-PCR)**	CTGCACGCCAAGATCGTC
**OsUBQ-5_F**	CTCGCCGACTACAACATCCA
**OsUBQ-5_R**	TCTTGGGCTTGGTGTACGTCTT
**Os18SrRNA_F**	CTACGTCCCTGCCCTTTGTACA
**Os18SrRNA_R**	ACACTTCACCGGACCATTCAA

Table S2. List of *Cis*-regulatory elements present in the promoter region of OsDOF27

	Factor or Site Name	Signal Sequence	Cis-elements	Description	Number of cis-elements
1	2SSEEDPROTBANAPA	CAAACAC	Storage protein; ABRE	Storage-protein gene promoters	2
2	-300CORE	TGTAAAG	Prolamin-box or P-box; DOF binding site	Binds with P-box binding factor	3
3	ABRECE1HVA22	TGCCACCGG	G-box; ABRE	ABA-responsive, Drought stress responsive	1
4	ABRELATERD1	ACGTG	ABRE-like sequence	Required for etiolation-induced expression of erd1 (early responsive to dehydration)	6
5	ABRERATCAL	MACGYGB	ABRE-related sequence	Ca2+ responsive	4
6	ACGTABREMOTIFA2OSEM	ACGTGKC	ABA-responsive	ABA-responsive	2
7	ACGTATERD1	ACGT	ACGT-sequence	Required for etiolation-induced expression of erd1 (early responsive to dehydration)	10
8	AGCBOXNPGLB	AGCCGCC	ERE; ERFs; Ethylene; GCC-box	Pathogen-responsive, ethylene-responsive element	2
9	ANAERO2CONSENSUS	AGCAGC	Cis-element in the promoter of anaerobic genes	Involved in fermentative pathway	7
10	ARR1AT	NGATT	ARR1-binding element	Present in the promoter of rice non-symbiotic hemoglobin-2 gene	8
11	BIHD1OS	TGTCA	Binding site of OsBIHD1	A rice BELL homeodomain transcription factor	1
12	CANBNNAPA	CNAACAC	Core of CA(n) element in storage protein gene in Brassica napus	Embryo and endosperm specific transcription of storage protein gene	3
13	CGACGOSAMY3	CGACG	CGACG element found in the GC-rich regions of rice Amy3D and Amy3E amylase gene	Recognized by Arabidopsis thaliana signal-responsive genes	11
14	CGCGBOXAT	VCGCGB	CGCG box	CuRE is found in Cyc6 and Cpx1 genes in Chylamydomonas	10
15	CURECORECR	GTAC	CuRE (Copper-responsive element)	DOF-Binding Site	6
16	DOFCOREZM	AAAG	DOF-Binding site	ABA-responsive	20
17	DPBFCOREDCDC3	ACACNNG	E-box	E-box of napA storage-protein gene of Brassica napus	3
18	EBOXBNNAPA	CANNTG	E-box	Important for Arabidopsis seed germination	8
19	GADOWNAT	ACGTGTC	Motif present in GA-downregualted d1 cluster (106 genes)	pathogen-responsive, ethylene-responsive element, jasmonate-responsive gene expression	2
20	GCCCORE	GCCGCC	GCC-box; ERE	GT1 stabilize TFIIA-TBP-DNA	10
21	GT1CONSENSUS	GRWAAW	Binding site in light-regulated genes	Important for GT-1 binding to box II of rbcS	13

	Factor or Site Name	Signal Sequence	Cis-elements	Description	Number of cis-elements
22	GT1CORE	GGTTAA	GT-1 motif	Plays role in pathogen and salt - induced SCaM -4 gene expression	1
23	GT1GMSCAM4	GAAAAA	GT-1 motif	Found in the promoter of tobacco late pollen gene g10	3
24	GTGANTG10	GTGA	GTGA motif	ABA responsiveness; Involved in cold induction of BN115 genefrom winter Brassica napus	10
25	LTRECOREATCOR15	CCGAC	Low temperature responsive element	Cis-element in the promoter of phenylpropanoid biosynthesis genes	3
26	MYBPLANT	MACCWAMC	MYB binding site	MYC recognition site found in the promoter of dehydration-responsive genes	5
27	MYCCONSENSUSAT	CANNTG	MYC binding site	Tissue specific expression; auxin induction	8
28	NTBBF1ARROLB	ACTTTA	NtBBF1 (Dof protein from tobacco) binding sites	Pollen-specific expression	3
29	POLLEN1LELAT52	AGAAA	Regulatory element for pollen specific activation	Involved in the induction of HSP70A gene	6
30	PRECONSCRHSP70A	SCGAYNRNNNNNNNNNNNNNNNHD	Plastid response element	Seed-specific expression; ABA-responsiveness	2
31	PROXBBNNAPA	CAAACACC	prox B (Proximal portion of B-box)	Gibberellin-responsiveness	2
32	PYRIMIDINEBOXOSRAMY1A	CCTTTT	Pyrimidine box found in rice alpha-amylase gene	Required for phytochrome regulation	1
33	REALPHALGLHCB21	AACCAA	RE-alpha element found in the Lemna gibba Lhcb1 gene promoter	Phloem specific gene expression	1
34	RNFG1OS	GATCATCGATC	RNFG1 binding site	Seed-specific expression; ABA responsiveness	1
35	RYREPEATBNNAPA	CATGCA	RY repeat found in the RY/G box of napA gene in Brassica napus	Sugar and ABA-responsiveness	3
36	SBOXATRBCS	CACCTCCA	S-box	Auxin-responsive element	1
37	SEBFCONSSTPR10A	YTGTCWC	Binding site of potato-silencing element binding factor	Light-responsive	1
38	SORLIP1AT	GCCAC	SORLIPS (Sequence overrepresented in in light induced promoter)	Auxin-responsive element	9
39	SURECOREATSULTR11	GAGAC	Sulfur responsive element (SURE)	Jasmonate-responsive	6
40	T/GBOXATPIN2	AACGTG	T/G-box found in tomato proteinase-II (pin2) and leucine aminopeptidase (LAP) gene	Guard cell-specific gene expression	2
41	TAAAGSTKST1	TAAAG	TAAAG motif found in the promoter of Solanum tuberosum	Light-responsiveness	6
42	TBOXATGAPB	ACTTTG	T-box	Fruit-specific expression	1
43	TGTCACACMCUCUMISIN	TGTCACA	TGTCACA motif	Binding site for WRKY proteins	1
44	WBBOXPCWRKY1	TTTGACY	WB-box		14

Table S3. Illustration of sequence parameters of OsDOF27 analysed by CIDER to evaluate the charge pattering and distribution, sequence composition and complexity

Parameters	Value	Description
f-	0.06227	Fraction of negative residues
f+	0.07692	Fraction of positive residues
FCR	0.13919	Fraction of charged residues
NCPR	0.01465	Net charge per residue
Kappa	0.30980	κ (charge patterning parameter
Omega	0.35440	Ω (charge/proline patterning parameter
Sigma	0.00154	⟨σ⟩, the charge asymmetry.
Delta	0.03962	δ value represents the square deviation of every blob σ value from the sequence's mean σ value
Max Delta	0.12789	δmax value represents the δ value associated with the maximally segregated sequence of the charge composition provided.
Hydropathy	4.18388	The Kyte-Doolittle hydropathy score for the sequence
Phase Plot Region	1Phase Plot Annotation: Globule/Tadpole	Location of protein on the Das-Pappu phase plot

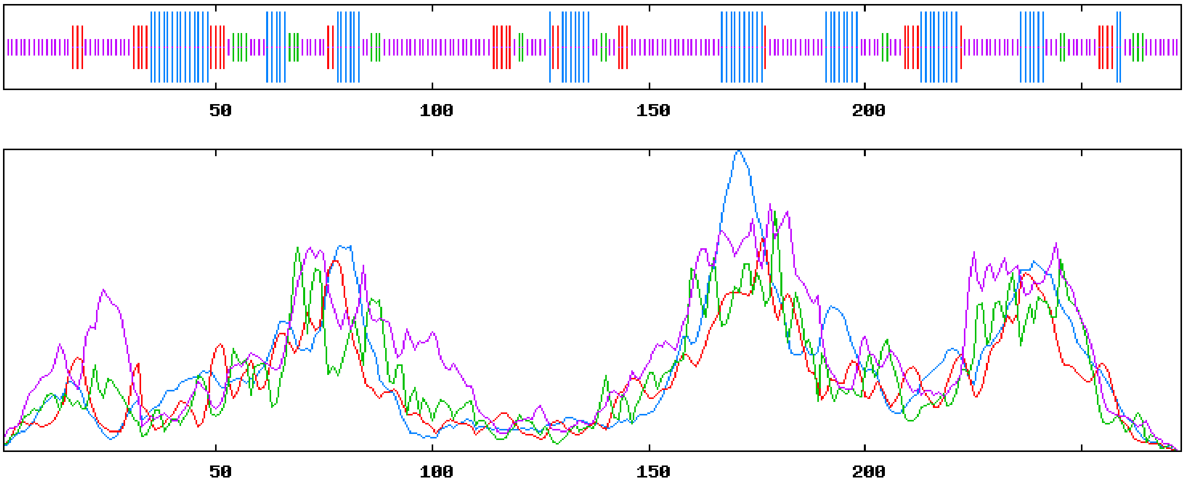


```
         10        20        30        40        50        60        70        80        90       100
          |         |         |         |         |         |         |         |         |         |
MSSPFLGSSSSSASSPLSYLTPPRPPPPPPPLLMVRHGLARWLTDLDDELMVFDDDLGVQGQGYAAAANGGIGGGGVEAVNAAAAPRQGGRHAGHPPLPR
ccccccccccccccccceeecccccccccccceeeehhhhhhhhhhhhheeeecttttcccchhhhhtttcccccceehhhhhhcctttccccccccccc
PPPRQCPRCGSANTKFCYYNNYSRTQPRYLCKACRRHWTEGGTLRDVPVGGGRKNSKRAAGGGKAGATASTAASAHVVAPAAAPPTSSSFPDLLRQMLMA
cccccccccccccceeeeecttccccchehhhhhhhcctttcceeeccccccccccccccccccccchhhhhhhhhhhecccccccccccccchhhhhhhhc
PATAGGGGGYSIDLTAWQQMAAFAAPPQAATGDVGGAVGAASTAAPDANCGGGGVQYWNGWLQDDMPGLDGSC
cccctttccceeeehhhhhhhhhecccccccccccccchhhhhhcccttcccccccceeeehhccttttcccccccc
```

Alpha helix (Hh) : 68 is 24.91%
3_{10} helix (Gg) : 0 is 0.00%
Pi helix (Ii) : 0 is 0.00%
Beta bridge (Bb) : 0 is 0.00%
Extended strand (Ee) : 33 is 12.09%
Beta turn (Tt) : 21 is 7.69%
Bend region (Ss) : 0 is 0.00%
Random coil (Cc) : 151 is 55.31%
Ambiguous states (?) : 0 is 0.00%
Other states : 0 is 0.00%

Figure S1. Secondary structure prediction of DOF27 sequence conducted by the online tool SOPMA to illustrate the secondary structures such as alpha helix, beta turn, extended strand, and random coil (https://npsa-prabi.ibcp.fr/cgi-bin/secpred_sopma.pl).

(A)

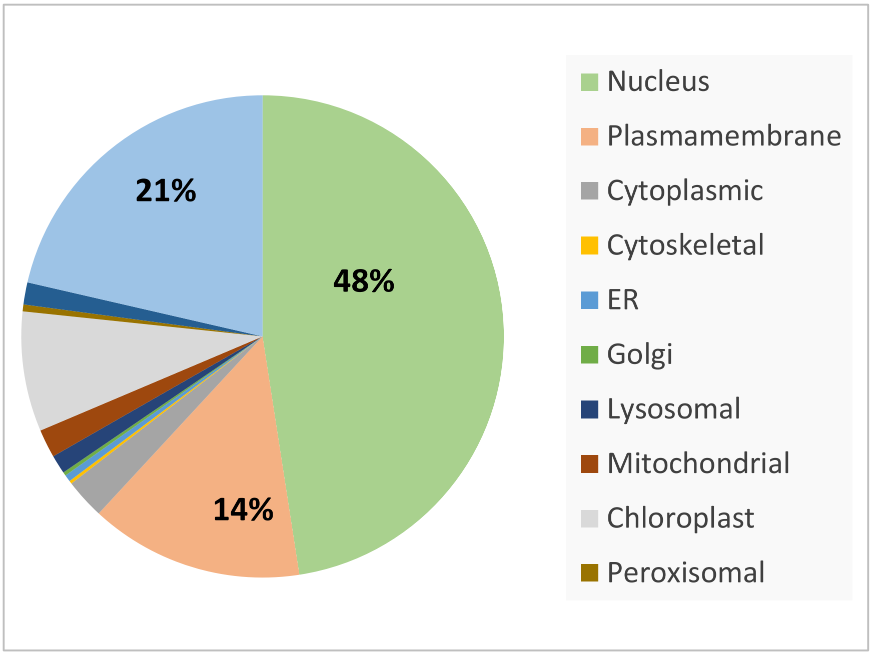

Nuclear	2.378
Plasma membrane	0.716
Cytoplasmic	0.134
Cytoskeletal	0.01
ER	0.023
Golgi	0.012
Lysosomal	0.063
Mitochondrial	0.095
Chloroplast	0.402
Peroxisomal	0.023
Vacuole	0.074
Extracellular	1.07

(B)

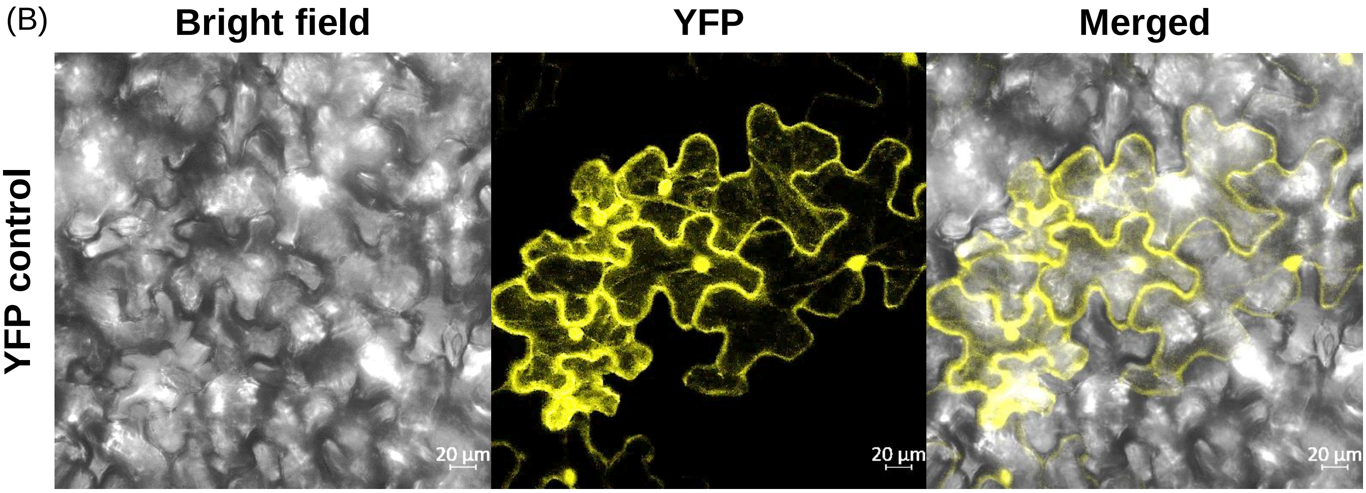

Figure S2 (A) An Insilco prediction of subcellular localization of OsDOF27 using online tools (http://cello.life.nctu.edu.tw/cello2go/). (B) Representative Nicotiana epidermal leaf cell transiently expressing YFP under the control of a 35s-CaMv (used as positive control). Scale bars = 20 µm. Confocal images of nicotiana leaves showing positive control (YFP signal) is targeted to entire cell, with an overlay of the YFP and, bright field (BF).

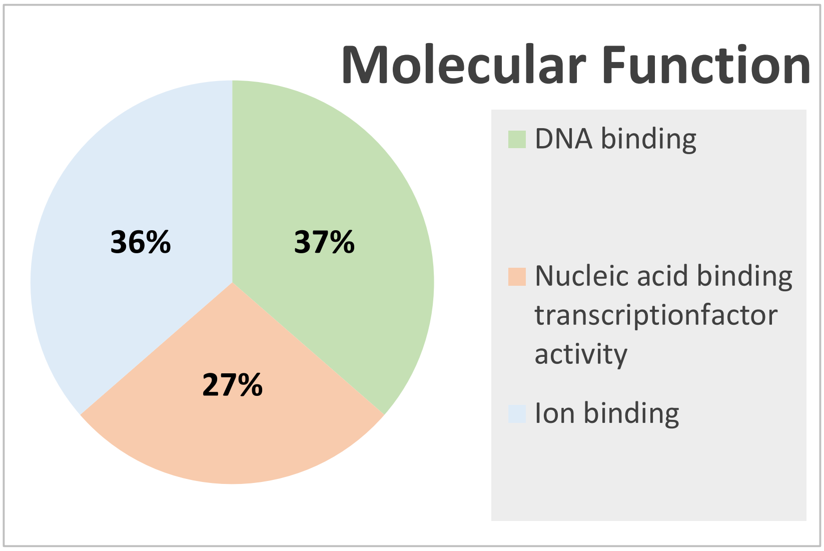

DNA binding	36.4 %
Nucleic acid binding transcription factor activity	27.19 %
Ion binding	36.4 %

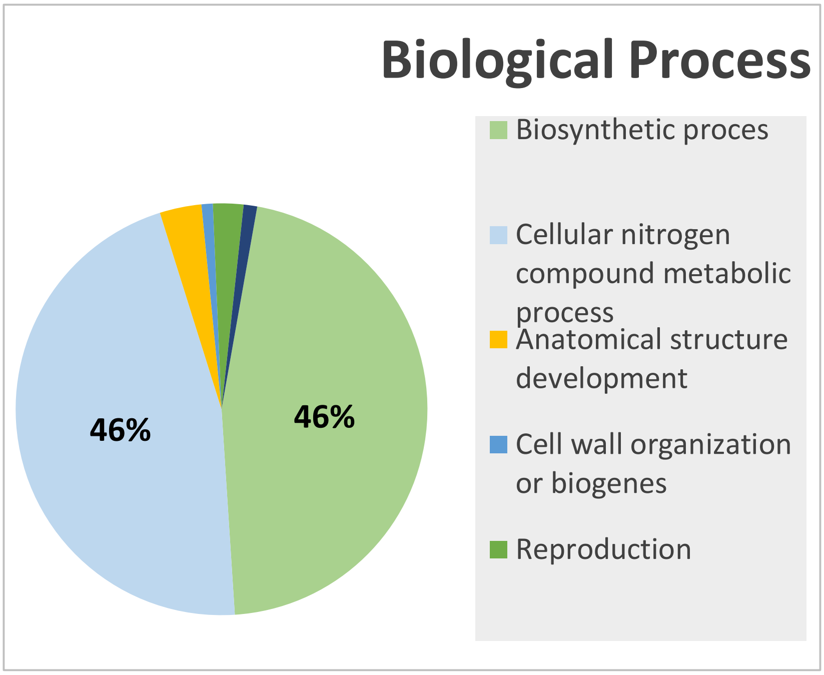

Biosynthetic process	45.79 %
Cellular nitrogen compound metabolic process	45.79 %
Anatomical structure development	3.25 %
Cell wall organization or biogenes	0.88 %
Reproduction	2.37 %
Embryo development	1.05 %

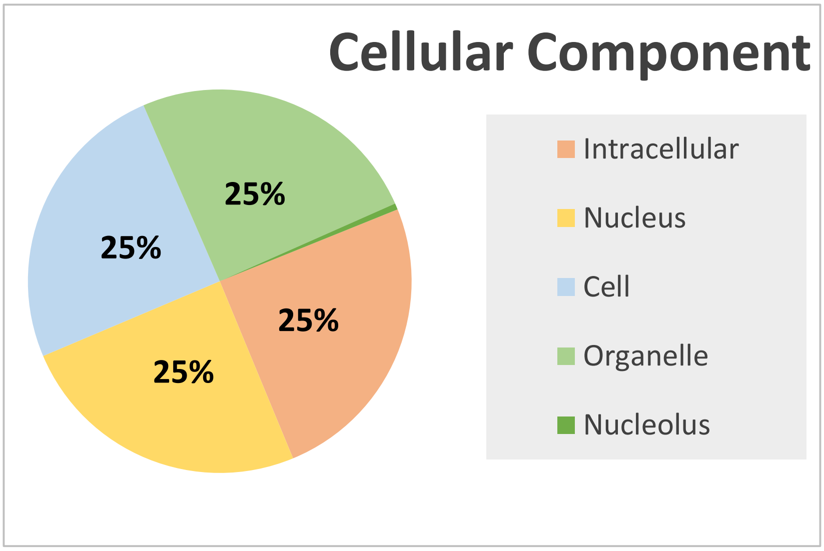

Cellular Component	
Intracellular	24.87 %
Nucleus	24.87 %
Cell	24.87 %
Organelle	24.87 %
Nucleolus	0.53 %

Figure S3 An Insilco analysis of functional Gene Ontology of OsDOF27 using online tools (http://cello.life.nctu.edu.tw/cello2go/).

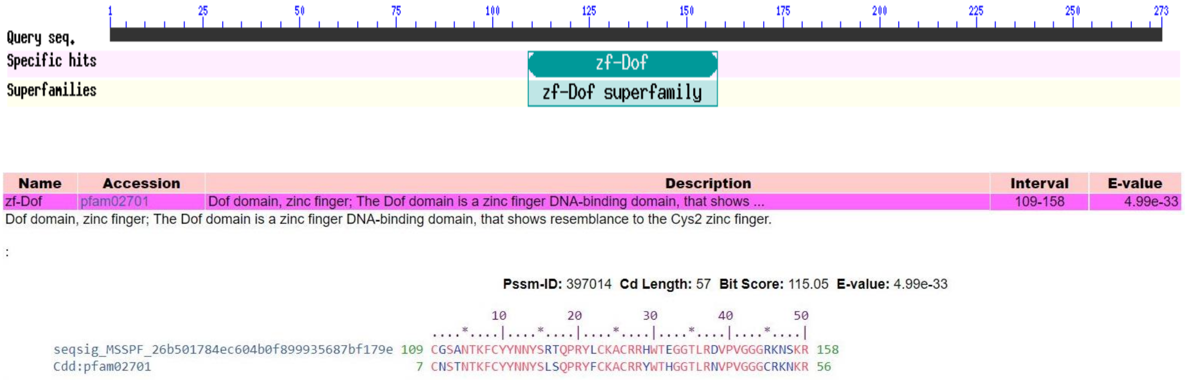

Figure S4: The CDD search profile of OsDOF27 protein sequence representing the 49 aa long Zin finger domain or DOF domain encompassing 109-158 aa . (https://www.ncbi.nlm.nih.gov/Structure/cdd/wrpsb.cgi)
